# Electrochemical Sensors for Sustainable Precision Agriculture—A Review

**DOI:** 10.3389/fchem.2022.848320

**Published:** 2022-05-09

**Authors:** Min-Yeong Kim, Kyu Hwan Lee

**Affiliations:** ^1^ Department of Electrochemistry, Korea Institute of Materials Science (KIMS), Changwon, South Korea; ^2^ Advanced Materials Engineering, Korea University of Science and Technology, Changwon, South Korea

**Keywords:** plant sensor, multi-detection, precision agriculture (PA), smart farm monitoring, electrochemical sensor (EC)

## Abstract

Greenhouse gases released by agriculture account for 19% of global greenhouse gas emission. Moreover, the abuse of pesticides and fertilizers is a fundamental cause of soil and water pollution. Finding sustainable countermeasures for these problems requires completely new approaches and the integration of knowledge. Precision agriculture (PA) is a technology that reduces environmental pollution with minimal input (e.g., fertilizer, herbicides, and pesticides) and maximize the production of high-quality crops by monitoring the conditions and environment of farmland and crops. However, the lack of data—a key technology for realizing PA—remains a major obstacle to the large-scale adoption of PA. Herein, we discuss important research issues, such as data managements and analysis for accurate decision-making, and specific data acquisition strategies. Moreover, we systematically review and discuss electrochemical sensors, including sensors that monitor the plant, soil, and environmental conditions that directly affect plant growth.

## 1 Introduction

Agriculture is facing a global crisis due to the depletion of natural resources, population growth, and climate change. According to UN projections, the world’s population could reach 9.15 billion by 2050—an increase of 2.25 billion from the current levels. Moreover, by 2050, global agricultural production and consumption are projected to increase by 60% compared to the levels in July 2005 (Alexandratos and Bruinsma, 2012) (Pawlak and Kołodziejczak, 2020). To meet this demand, agricultural production can be increased by improving cultivation practices, expanding farm sizes, and utilizing automation technology. However, a sustainable and comprehensive method for increasing crop yields and improving the associated environmental pollution has not been clearly presented (Bailey-Serres et al., 2019). Therefore, it is important to develop a model that allows environmental conservation and improved productivity in the agricultural field. Such a model should also help deal with challenges (such as an insufficient labor force), reduce uncertainty with systematic management, and achieve eco-friendliness and economical benefit at the same time (Yin et al., 2021).

Precision agriculture (PA) is a smart and sustainable system that maximizers agricultural yields and minimizes environmental side effects by improving resource utilization (Pedersen and Lind, 2017). Recently, the 4th industrial revolution has seen the rapid development of big data, cloud computing, drone technology, and the internet of things (IoT). (Jawad et al., 2017). Ideally, PA would involve building cloud-based big data platforms through IoT-based data collection, which would held optimize prediction and allow customized prescriptions based on artificial intelligence (AI). Moreover, it may be possible to optimize work through the use of intelligent agricultural machinery or agricultural robots that can provide real-time information to farmers through mobile devices (Ratnaparkhi et al., 2020). This convergence of agriculture and communication technology can help develop eco-friendly farming methods through the automation of agricultural machinery using advanced technology. It can also improve the predictability of crop growth and climatic conditions through sensor and information processing technology, and can help develop new fertilization technology. Therefore, PA is expected to be a systematic alternative to the aging rural workforce, provide imbalance in supply and demand for agricultural products, and help mitigate agriculture-related environmental pollution (Marios and Georgiou, 2017).

For the stable performance of an intelligent and sustainable agricultural system, it is essential to develop a monitoring system that provides key information for building a site-specific database of crop growth and soil and environmental conditions (Shafi et al., 2019). Traditionally, the properties of the soil and growing plugs are measured *in situ* through visual inspection and experience. In addition, factors such as salinity, water stress, viral and bacterial diseases, and growth inhibition by weeds and pests are evaluated through visual inspection and laboratory analysis (Martinelli et al., 2015). However, these conventional strategies may not be sufficiently accurate and prompt. An efficient PA system requires novel technologies that can monitor real-time information in a prompt and accurate manner. In particular, developing a systematic diagnostic technique by analyzing the compositions of crop sap and nutrient solution is more important than monitoring physical factors, such as the size and apparent color of leaves or fruits.

Of the aforementioned technologies, advanced sensing systems that monitor crop growth and soil and environmental conditions are the most important, as they collect data that is important for operator decision-making, especially when crop growth conditions vary significantly across seasons and locations (de Araujo Zanella et al., 2020). A wide range of analytical methods such as chemiluminescence, spectrophotometry, fluorescence, and chromatography have been used as many approaches for monitoring plant growth in agriculture. However, this traditional analysis method is not suitable for on-site monitoring because the pretreatment is complicated and expensive equipment and highly skilled technicians are required. On the other hand, the electrochemical detection system is particularly useful as it is a lightweight, portable device that is convenient to use, has a fast response (Pandikumar and Rameshkumar, 2018). Moreover, electrochemical sensor technology has a smaller sample amount, higher measurement accuracy, and less influence from the surrounding environment. The fact that measurement is simple as well as real-time detection is a critical advantage in the agricultural field where real-time monitoring is important. While the existing analysis methods analyze the current results, the electrochemical sensor can monitor changes in the growth state of plants in real time, so it is possible to respond in a preventive process. When this electrochemical sensor technology is commercialized in earnest, monitoring of plant growth information, early diagnosis of disease, and environmental pollution detection can be performed very accurately and prompt with one portable device. Electrochemical sensors that detect target materials in various ways can be applied to wide fields such as industry, medicine, agriculture, and the environment, and it is expected that active research in the agricultural field will proceed in the future (Lu et al., 2020) (Lan et al., 2020) (Im et al., 2018) (J. J. Kim et al., 2020) (Lee et al., 2021).

In this review, we summarize the current state and recent developments in PA, and focus on the key element of an electrochemical sensing system capable of accumulating specific information during various stages of plant growth. In particular, we focus on monitoring techniques that use phytochemicals—rather than physical parameters, such as plant size and color—for the early diagnosis of disease and stress. Moreover, we discuss research trends in plant sensors and PA, problems faced by agricultural sensors, specific strategies for building big data infrastructures, and future directions and challenges of PA.

## 2 New Agricultural Trend

### 2.1 Precision Agriculture

PA involves a convergence of agricultural science, information and communication technology, and analytical technology, and uses advanced sensors and tools to improve crop yields and support management decisions (Khanna and Kaur, 2019). PA aims to reduce environmental pollution through minimal input (e.g., fertilizers, herbicides, and pesticides), and maximize the production of high-quality crops by monitoring the environment and conditions of farmland and crops (McBratney et al., 2005). PA proceeds in four steps: 1) observation, 2) prescription, 3) agricultural work, and 4) result analysis. The 1st stage involves monitoring to collect data on farmland, crop growth, and agricultural machinery. These data are analyzed using AI and machine learning techniques, and the results are used to make appropriate prescriptions in the 2nd stage. The prescriptions are used as the input conditions for the agricultural work in the 3rd stage, and the results of the agricultural work are used for the analysis in the 4th stage. This four-step precision farming cycle leads to the creation of a comprehensive database, and the accumulated agricultural data are used as basic data for more efficient production of high-quality crops. The 1st stage (observation) involves data collection through various sensors; therefore, sensor technology for data collection in the field is of primary importance. Examples of sensory technologies for agricultural data include image and electrochemical sensors. Apart from image sensing, the most common agricultural sensors (Abbasi et al., 2014) for crop-related parameters are listed in Table 1. Most image and electrochemical sensors are devices that measure parameters related to physical quantities, such as images, temperature, humidity, and electrical conductivity (Martinière et al., 2013). Recent research has focused on sensors that can identify the growth stage of plants and determine the presence or absence of disease using machine learning techniques on digital images (Sadeghi-Tehran et al., 2017).

**TABLE 1:** Common sensors and detection parameters in agriculture (Abbasi et al., 2014).

	Sensor name	Parameters monitored
1	ECH2O soil moisture sensor	Soil temperature, soil moisture, conductivity
2	Hydra probe II soil sensor	Soil temperature, salinity level, soil moisture, conductivity
3	MP 406 soil moisture sensor	Soil temperature, soil moisture
4	EC sensor (EC250)	Soil temperature, salinity level, soil moisture, conductivity
5	Pogo portable soil sensor	Soil temperature, soil moisture
6	237 leaf wetness sensor	Plant moisture, plant wetness, plant temperature
7	YSI 6025 chlorophyll sensor	Photosynthesis
8	TT4 multi-sensor thermocouple	Plant moisture, plant temperature
9	TPS-2 portable photosynthesis	Photosynthesis, plant moisture, CO_2_
10	CM-100 Compact weather sensor	Air temperature, air humidity, wind speed, air pressure
11	XFAM-115KPASR	Air temperature, air pressure, air humidity

**FIGURE 1:**
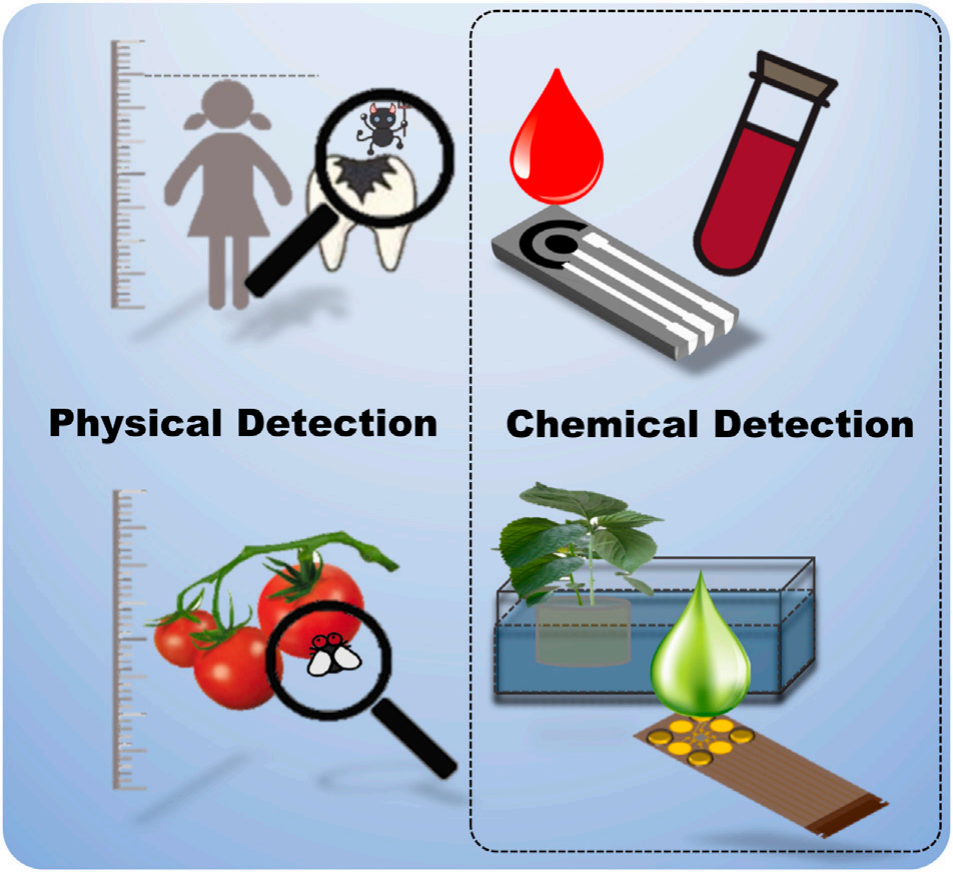
Comparison of physical and chemical sensors for the examination of health in humans and plants.

Physical sensors alone are insufficient for capturing all the information necessary for optimal crop growth (Duhan et al., 2017). As an example, there are various ways to predict disease in a person through a medical check-up. One method is to judge their health condition by observing their external condition and physical information such as height, weight, heart rate, physical strength, blood pressure, and appearance (Figure 1). However, a more detailed internal inspection is possible through blood tests that assess hormones, blood composition, blood sugar, and various cancer targeting components. Clearly, the latter method provides more specific health-related information, and allows the prediction of various diseases (Voke et al., 2021). Therefore, it is necessary to approach PA from a causal point of view by developing chemical sensors that can monitor nutrient compositions of plant sap and hydroponic nutrient solutions, which can directly determine crop growth information.

### 2.2 Electrochemical Sensors for Plants

**FIGURE 2:**
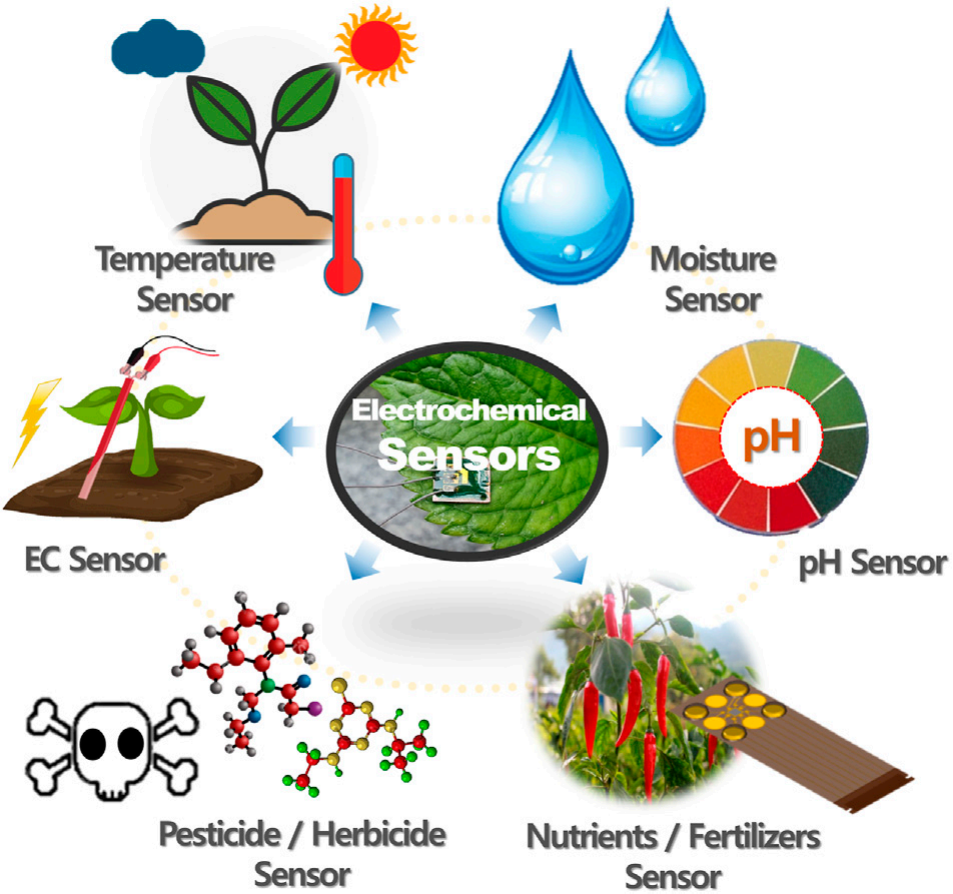
The electrochemical sensor for monitoring plant health in precision agriculture.

Plant growth is influenced by complex and mutual relationships, including adequate nutrition, various environmental conditions, photosynthesis, respiration, pests, and diseases (Schans and Arntzen, 1991). To ensure sufficient agricultural production, it is essential to monitor the factors affecting plant growth in real time. In this section, we discuss the development of plant sensors based on electrochemical systems and the various efforts to create them. Figure 2 illustrates the various types of electrochemical sensors for monitoring plant growth, and Table 2 summarizes the electrochemical sensors for monitoring plant signals. Moreover, Table 3 shows the nutrient concentration table of commonly used hydroponics. Electrochemical sensors have the potential to systematically monitor plant health and provide early diagnoses of disease and stress. Thus, these can replace conventional approaches (such as image-sensing technology) for monitoring plant health.

**TABLE 2:** Electrochemical system-based plant signal sensors.

Target materials	Sensing materials	Detection signals	Applications	References
Humidity sensor	GO	Capacitance change	Plant leaf water detection	Lan et al. (2020)
Humidity sensor	polyimide	Capacitance change	Plant leaf water detection	Im et al. (2018)
NO_2_ sensor	Silver/rGO	Resistance change	Toxic gas detection	Li et al. (2018)
VOC sensor	CNT/graphite	Resistance change	Gas detection from the plant	Li et al. (2021)
VOC sensor	rGO/AnNPs	Resistance change	Plant health monitoring	Calisgan et al. (2020)
O_3_ sensor	PEDOT-Cl	Impedance and phase change	O_3_ damage detection in plant	Kim et al. (2020)
Pesticide sensor	LIG-OHP/AuNPS	Current change	*In-situ* analysis of pesticide	Zhao et al. (2020)
Nutrient sensor	rGO aerogel/ISE	Potential change	Plant sap monitoring	Kim et al. (2021)
Nutrient sensor	LIG/ISE	Potential change	Nitrogen sensing in soil	Garland et al. (2018)

GO, graphene oxide; rGO, reduced graphene oxide; CNT, carbon nanotube; AuNPS, gold nanoparticle; PEDOT, poly (3,4-ethylenedioxythiophene); LIG, Lase-induced graphene; ISE, ion selective electrode; VOC, volatile organic compounds.

**TABLE 3:** Concentration table of commonly used Yamazaki lettuce and Enshi strawberry nutrient solutions (Liu and Huang, 2019).

Element	Yamazaki conc. (mg L^−1^)	Enshi conc. (mg L^−1^)
Nitrogen	92.2	245.8
Potassium	156.2	312.5
Phosphorus	15.4	41.8
Calcium	40.1	161.3
Magnesium	12.1	49.3
Sulfur	16.1	65.4
Iron	2.4	3.8
Sodium	1.0	1.6
Boron	2.8	0.52
Manganese	0.14	0.46
Zinc	0.02	0.05
Copper	0.01	0.012
Molybdenum	0.0052	0.008

#### 2.2.1 Plant Nutrient Sensors

**FIGURE 3:**
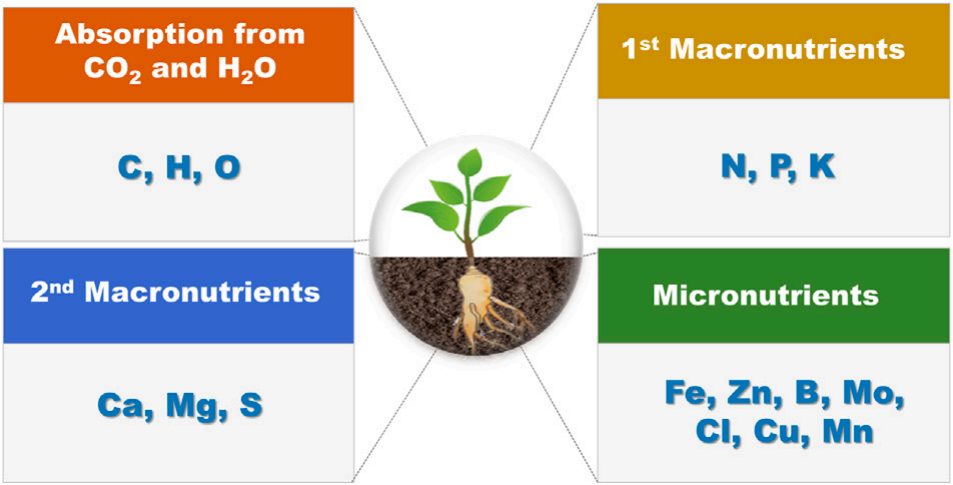
Types of essential nutrients needed for plant growth.

Inorganic nutrients (fertilizers) are essential for plant growth, and are even more important for high crop yields in agriculture. Among the various inorganic nutrients in plants, 16 are known to be essential elements that are required by crops (Figure 3). (Muñoz-Huerta et al., 2013) The essential nutrients necessary for plant metabolism include carbon, hydrogen, and oxygen (which can be absorbed from water and carbon dioxide), macronutrients (e.g., nitrogen, potassium, calcium, phosphoric acid, sulfur, and magnesium), and micronutrients [e.g., chlorine, boron, iron, manganese, zinc, copper, nickel, and molybdenum. (Kashyap and Kumar, 2021)] The demand for these ions differs between plant types and growth stages, and the ions interact intricately through various absorption mechanisms in plant roots (Gieling et al., 2005) (Guignard et al., 2017). Therefore, accurately monitoring these fertilizers can reduce the waste of fertilizers and protect the environment, which can bring about various economic effects (Kashyap and Kumar, 2021), (Yin et al., 2021). A representative method for analyzing nutrients in crops *in situ* is to use an ion-selective electrode (ISE) (Cheong et al., 2021).

The ISE measures the potential difference generated by the binding of a target ion to the selective membrane using a membrane that selectively reacts with a specific analyte. Unlike other analytical methods, ISE measurements can be conducted on site without the need for pretreatment. Therefore, it is a simple and rapid method to analyzes ions in environmental, water, food, pharmaceutical, and biological samples (Shao et al., 2020). An ISE can be created by attaching an electrode polymer membrane—comprising an ion-sensitive material (ionophore) that reacts with the ionic material—to the electrode surface. Ionophore-doped polymeric membrane-based ISEs exhibit excellent processability, and enable the detection of various analytes (Chen et al., 2018). Conventional membrane-based ISEs are liquid contact-type ISEs with an internal filling solution on an electrode, designed as a glass membrane. Liquid-contact ISEs are relatively stable in a research environment, and are already commercially available; however, they are difficult to maintain, and miniaturization is disadvantageous because of the presence of an internal liquid. Compared to liquid-contact ISEs, solid-contact ISEs are stable, simple to manufacture, and can be miniaturized (due to the absence of an internal liquid). Recently, solid-contact ISEs have shown excellent performance improvements when various solid-contact functional materials were applied as ion-to-electron converters (Hu et al., 2016). These instruments can be miniaturized and require simple assembly. Moreover, the use of disposable screen-printed electrode (SPE) substrates in an economical way presents the advantages of durability and reproducibility.

**FIGURE 4:**
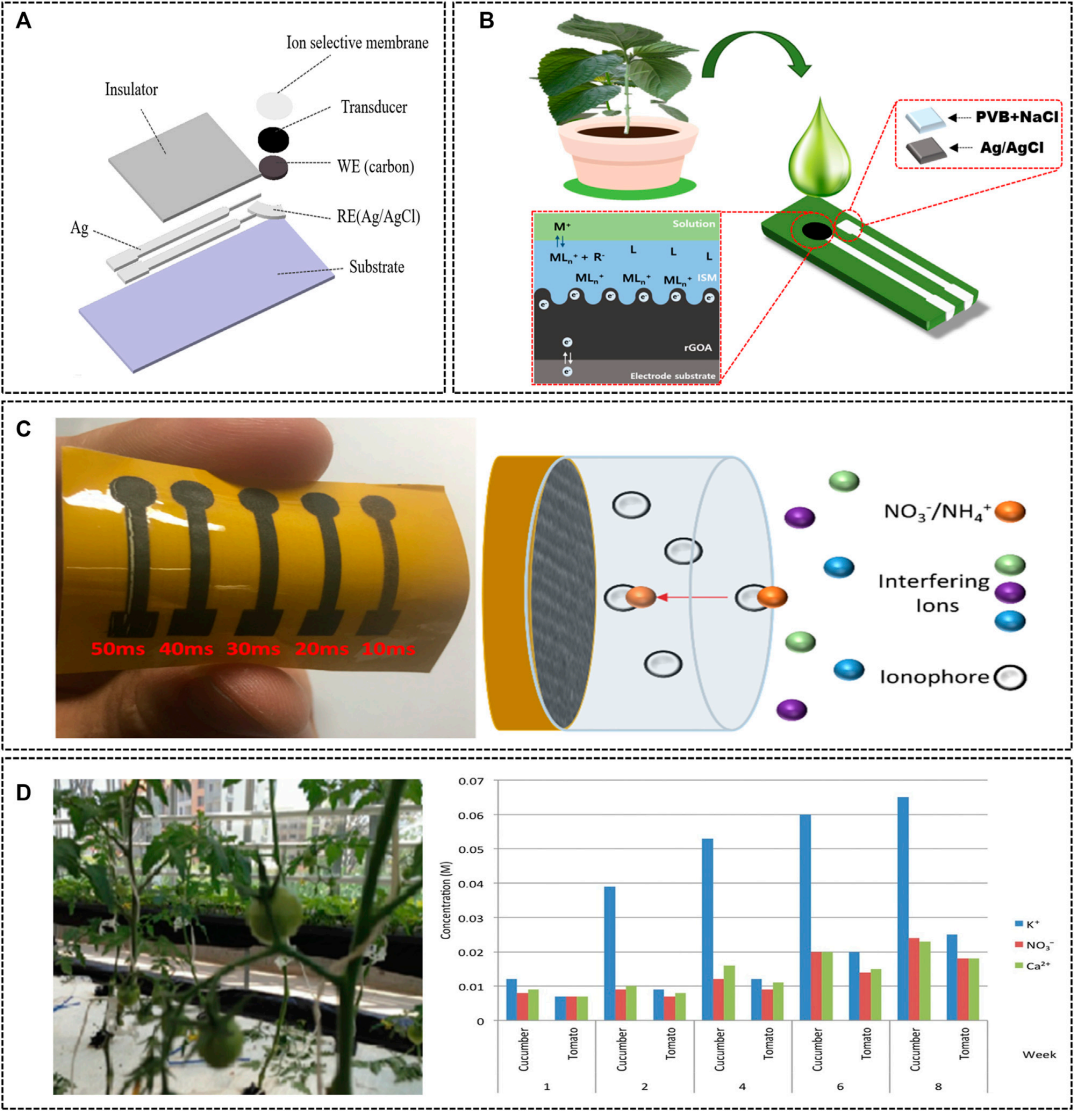
**(A)** Schematic representation of the SC-ISE based SPE (M.-Y. Kim et al., 2021). Copyright ^©^ 2021 (Elsevier). **(B)** Reduced graphene oxide aerogel-based SC-ISE for Ca^2+^ and NO_3_^−^ detection in the plant sap (M.-Y. Kim et al., 2021). Copyright ^©^ 2021 (Elsevier). **(C)** Laser-induced graphene-based SC-ISE for the determination of NO_3_^−^ and NH_4_^+^ levels in the soil sample (Garland et al., 2018). Copyright ^©^ 2018 (American Chemical Society). **(D)** The hydroponic greenhouse control systems (left) and mineral uptake by cucumber and tomato plants in nutrition reservoirs (right) (Vardar et al., 2015). Copyright ^©^ 2015 (Wiley-VCH).

Recent studies have reported the detection of nutrients in plant sap, soil, and nutrient solutions using a potentiometric ion sensor based on solid contact-ion selective electrode (SC-ISE). Kim et al. reported an increase in the stability of the sensor by using a disposable SPE-based reduced graphene oxide aerogel (rGOA) as an electron-ion converter to measure Ca^2+^ and NO_3_^−^ in plant sap (Figure 4) (M.-Y. Kim et al., 2021). Using rGOA with high capacitance as an electron-ion converter, the sensor showed high stability. In addition, the sensor measured Ca^2+^ and NO_3_^−^ in the sap of perilla leaves, and the results displayed high performance similar to those of the ICP and IC methods. Artigas et al. developed a sensor probe incorporating a screen-printed electrode-based nitrate-selective electrochemical sensor equipped with a copper plate reference electrode (Artigas et al., 2003). This sensor has shown promising results in monitoring the ionic state of inorganic nitrogen in soil using the potentiometric method. Using laser-induced graphene (LIG) as the solid electrode layer of SC-ISE also reduces toxicity, and requires a simple manufacturing procedure (Garland et al., 2018). In this method, a flexible LIG electrode is attached to polyimide (PI) film through a manufacturing procedure free of toxic chemicals. The LIG-based SC-ISE has been used to measure NO_3_^−^ and NH_4_^+^ levels in soil slurry, and showed excellent detection abilities. Jiang et al. developed an ion sensor including an ion-selective electrode (that uses the potentiometric method) to measure the concentrations of H_2_PO_4_^−^ and NO_3_^−^ in the growth medium of rice plants (Jiang et al., 2017). In this device, an ion-selective electrode is attached to the bottom of a poly (methyl methacrylate) (PMMA) chamber filled with growth medium, and a plastic mesh is mounted on the top of the sensor to prevent contact with plant roots. This potentiometric sensor successfully quantified H_2_PO_4_^−^ and NO_3_^−^ levels in plants for 2 weeks, and demonstrated the possibility of monitoring the nutrient utilization efficiency of plants in real time. Vardar et al. used SC-ISE to detect ions in a hydroponic nutrient solution (Vardar et al., 2015). NO_3_^−^, K^+^, and Ca^2+^ sensors were prepared using the previously described ion-selective membrane, and these sensors could detect changes in the concentration of ions in hydroponic nutrient solutions according to the growth stage of crops. Monitoring the levels of NO_3_^−^, K^+^, and Ca^2+^ in cucumber and tomato crops revealed that tomatoes consumed less nitrate than cucumbers during the first 2 months of growth, and that potassium intake was higher than that of other nutrients. The results of these studies indicate that the types and amounts of nutrients absorbed by plants differ between crop types, growth stages, and seasons. Kim et al. reported the direct measurement of NO_3_-N, K, and Ca concentrations using ISE in a paprika hydroponic nutrient solution (H.-J. Kim et al., 2013). An ISE array coupled with a computer-controlled measurement system has been shown to monitor the ion concentrations of nutrient solutions over time. Therefore, systematically generating a nutrient absorption mechanism and prescription map for each crop and growth stage and help improve the possibility and performance of PA.

#### 2.2.2 Plant Hormone Sensors

Measuring phytohormones *in situ* in agricultural fields is a remarkable technique, as phytohormones play a crucial role in regulating physiological processes and defensive signals in plants (Giraldo et al., 2019). Monitoring nutrients and crucial plant physiological parameters *in vivo* is a key component of PA. Plant hormones are important molecular aggregates that regulate plant growth, development, and other physiological processes (Santner and Estelle, 2009). Because these plant hormones are directly related to crop yields, a precise monitoring technology is required to measure phytohormones (Giraldo et al., 2019). Plants exhibit hormonal changes due to various environmental stresses, and these changes are associated with different electrochemical patterns (Kinpara et al., 2001). Therefore, the technology to monitor plant hormones can help predict various diseases and stress responses, such as responses to drought, salinity, and pest stress (Suzuki et al., 2014).

**FIGURE 5:**
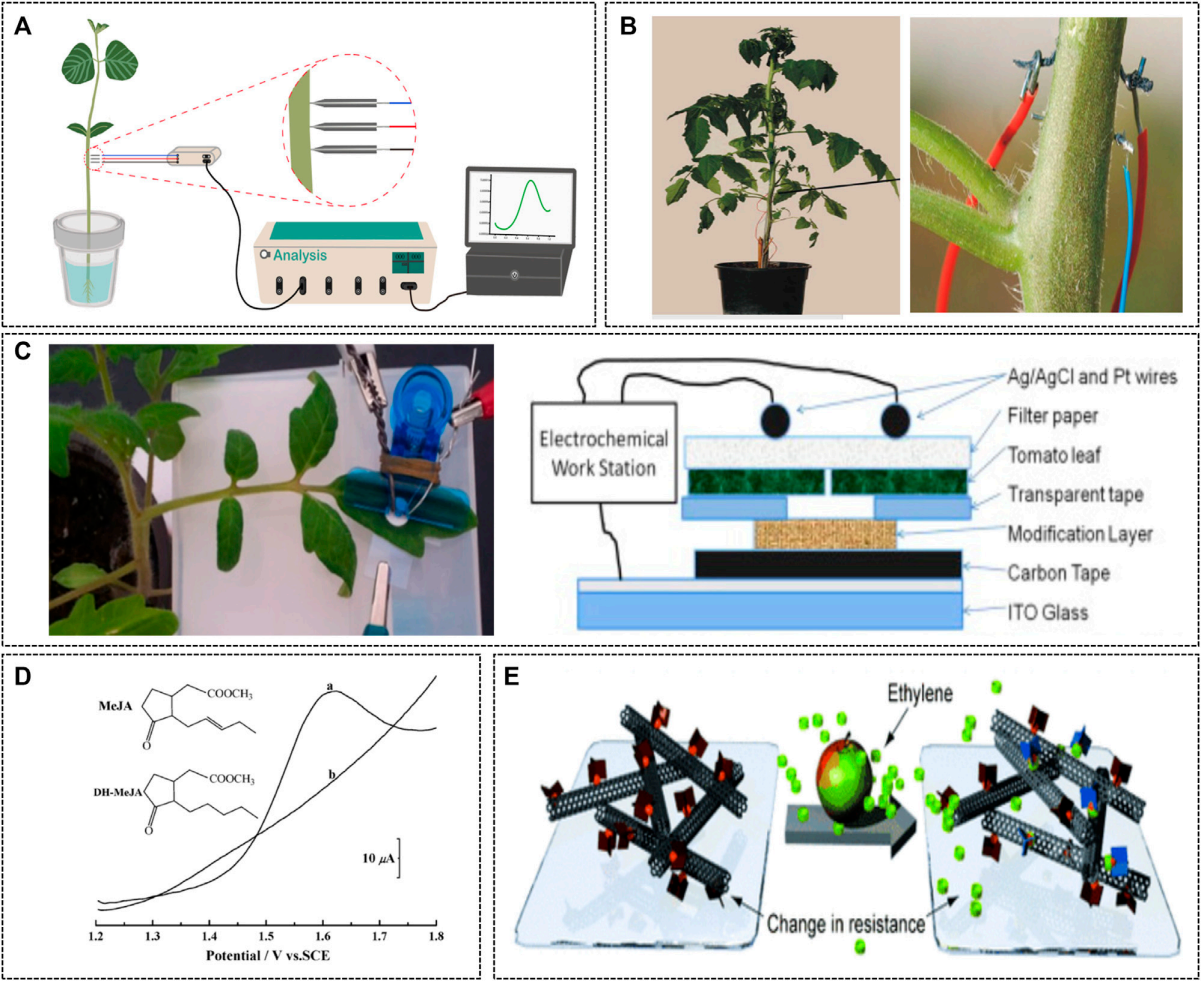
Electrochemical sensor for monitoring plants hormones. **(A)** A schematic illustration of *in vivo* determination of auxins in soybean seedlings (H. Li et al., 2019). Copyright ^©^ 2019 (Elsevier). **(B)** The textile sensor device is implanted in a tomato plant stem for monitoring variations in the solute content of the plant sap (Coppedè et al., 2017). Copyright ^©^ 2017 (Nature). **(C)** A paper-based electroanalytical device (left) and its cross-section view (right) (Sun et al., 2014). Copyright ^©^ 2014 (Elsevier). **(D)** Square-wave voltammograms of methyl jasmonate (MeJA) and DH-MeJA (Gan et al., 2010). Copyright ^©^ 2010 (American Chemical Society). **(E)** Schematic image of ethylene sensing using a chemoresistive sensor (Esser et al., 2012). Copyright ^©^ 2012 (Wiley-VCH).

Auxins play a central role in the regulation of plant growth and development. Substances that promote plant growth, 3-indole acetic acid (IAA), and other natural substances with the same function are collectively called auxins (Adamowski and Friml, 2015). Auxins increase the permeability of cell membranes and promote the growth of individual cells by promoting cell division, irregular root formation, and flower bud formation (Sun et al., 2017). Auxins are produced in new leaves and are transferred to the roots (in the direction of gravity) to promote root development. Recently, an electrode using a stainless steel (SS) wire was developed for the detection of IAA (a representative plant growth hormone) (H. Li et al., 2019). The proposed electrochemical microsensor showed a low detection limit of 43 pg ml^−1^, and successfully detected IAA *in vivo* in the stems of soybean seedlings under saline stress conditions (Figure 5). Coppedè et al. proposed a biomimetic fiber-based electrochemical biosensor that could be directly inserted into plant tissues (Coppedè et al., 2017). This sensor can monitor changes in the solute content of plant sap *in vivo* in real time. These changes can indicate signs of stress, monitoring which can help improve plant growth. Moreover, these data can be used for data-building in PA. Apart from auxins, salicylic acid (SA) is another type of plant hormone that regulates central signals for the physiological behaviors of plants (Sun et al., 2014). A disposable working electrode was developed by coating a carbon tape with a mixture of multi-walled carbon nanotubes and Nafiion, and this was used to detect salicylic acid released from tomato leaves. The electrochemical reaction of salicylic acid was greatly enhanced by treating the modified carbon tape electrode with oxygen plasma. The results showed that the amount of salicylic acid released from tomato leaves was statistically different between normal and diseased tomato leaves infected with *Botrytis cinerea*. Therefore, this electrode could potentially detect plant conditions under biotic and environmental stresses by quantifying salicylic acid at nanogram levels in real time.

Jasmonic acid and ethylene are two representative phytohormones that respond to environmental changes (Xu et al., 2020). A previous study has reported the direct electrochemical determination of methyl jasmonate (MeJA) using a nano-montmorillonite modified glassy carbon electrode (nano-MMT/GCE) (Gan et al., 2010). This electrode exhibited a sensitive electrochemical signal for the direct oxidation of MeJA, and this method was applied to the detection of MeJA released from wheat spikelet samples. Ethylene, the smallest phytohormone, plays a role in initiating the ripening of fruits, is involved in several plant developmental processes, and promotes seed germination, flowering, and senescence of leaves and flowers. Ethylene is difficult to detect selectively because of its small size and nonpolar chemical properties. However, ethylene was selectively detected using carbon nanotubes and a copper (I) complex one based on a fluorinated tris(pyrazolyl)borate ligand (Esser et al., 2012). The copper (I) complex one combines with ethylene to form complex 2, which shows reduced interaction with the surface of the single walled carbon nanotube (SWCNT). This change causes a corresponding change in the resistance of the SWNT network. These changes were measured as electrochemical signals and used for the detection of ethylene. In conclusion, the real-time detection of various plant hormones can help monitor plant physiological changes under environmental and biological stress, and thus help maintain the optimal conditions for plant growth.

#### 2.2.3 Other Sensor for Plants

**FIGURE 6:**
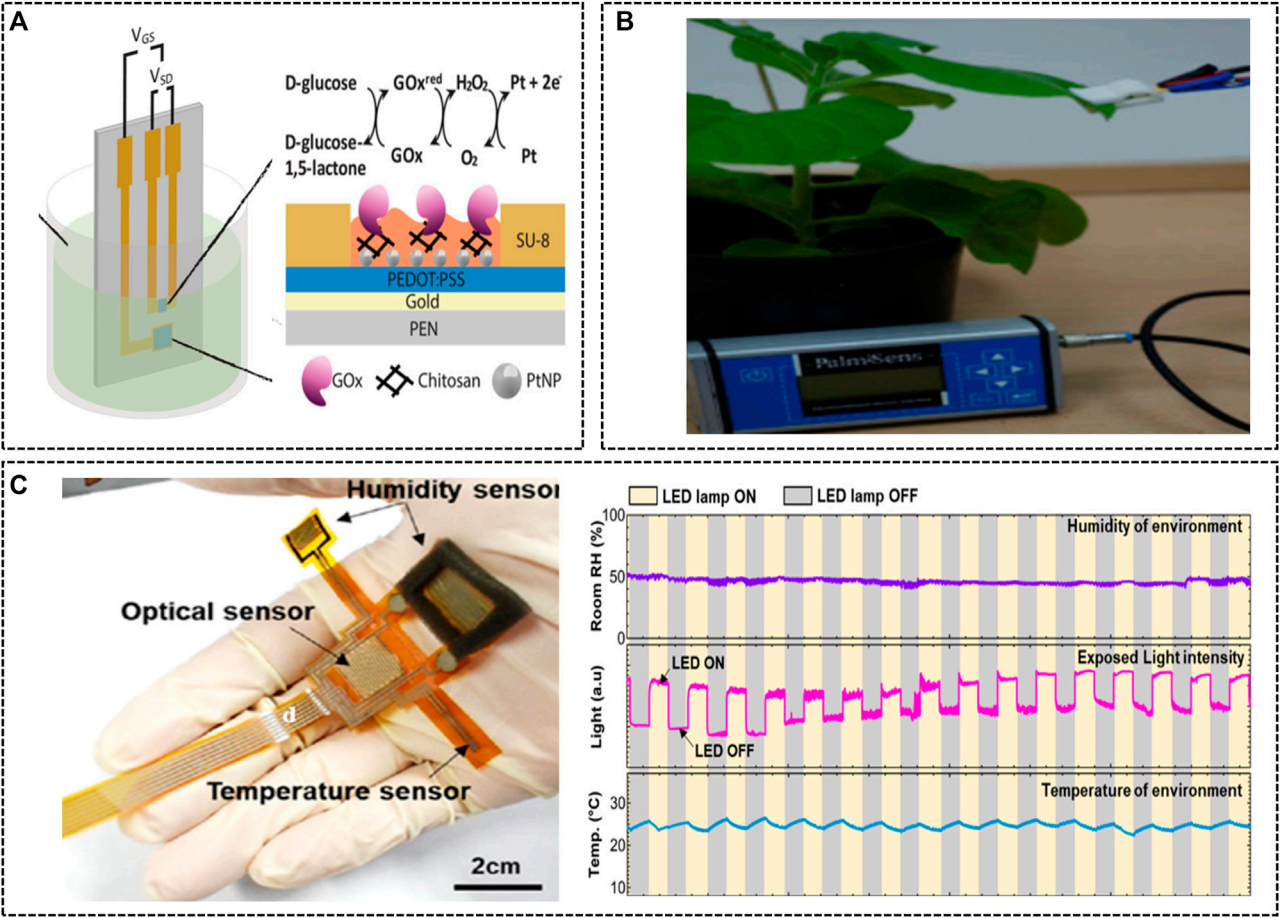
Electrochemical plant sensors. **(A)** Overview of the experimental design for the detection of glucose in isolated chloroplasts (Diacci et al., 2020). Copyright ^©^ 2020 (Wiley-VCH). **(B)** A 3D-printed chip holder containing a hand-held potentiostat and sensor chip (Pandey et al., 2018). Copyright ^©^ 2018 (Elsevier). **(C)** A multimodal flexible plant sensor integrated with humidity, optical, and temperature sensors (left) and thee results of transpiration monitoring using a multimodal flexible sensor device for over 350 h (right) (Lu et al., 2020). Copyright ^©^ 2020 (American Chemical Society).

In addition to nutrients and plant hormones, a variety of other parameters can be used to monitor plant growth status and physiological conditions. Plants have evolved signaling networks that trigger appropriate physiological processes as a response to changing environmental conditions (Lee et al., 2021). Detecting plant stress markers based on chemical properties (such as plant signaling molecules) may help diagnose specific environmental or biological stresses in plants. Electrochemical biosensors using organic electrochemical transistors (OECTs) are suitable systems for real-time monitoring of biological processes. The direct bonding between the OECT channel and the electrolyte allows for signal amplification and a prompt sensor response, while maintaining a close interface with biological systems. An OECT sensor that uses glucose oxidase cross-linked with a chitosan matrix as a sensing mechanism has been reported to detect glucose levels from the chloroplast (a plant organelle that synthesizes glucose) (Diacci et al., 2020). Glucose synthesized from chloroplasts was monitored in real time at two distinct plant metabolic stages (Figure 6), and these results showed improvements over existing methods. Electrochemical sensors for glucose detection have been developed using various materials (Naikoo et al., 2021). These monitoring techniques are important because the production of glucose in chloroplasts is directly related to the yield and sugar content of fruits (Naikoo et al., 2021). Moreover, researchers have developed a technique for the real-time monitoring of β-glucuronidase (GUS) expression in transgenic tobacco plants using biomarker activity (Pandey et al., 2018). Microchip electrodes were designed with a three-electrode system using the chronoamperometric technique, and were used to convert the expression of GUS enzymes in Msk8 tomato cells and BY2 tobacco cells into measurable current signals. The detection sensitivity of the GUS expressed in tomato cells was 0.076 mA/mM cm^2^, and the detection limit was calculated to be 0.1 mM. The interaction and detection of these biological markers in plants will provide new opportunities for the detection of plant growth status in PA. Moreover, multifunctional plant-wearable sensing devices have been developed using advanced nanoelectronic technology to monitor potential health issues of plants. A multimodal flexible sensor system for monitoring changes in plant growth status has been proposed using stacked ZnIn2S4 (ZIS) nanosheets as the sensing medium (Lu et al., 2020). The ZIS-based flexible sensor detected the illuminance and monitored the humidity in the vicinity of the leaves (i.e., where plant transpiration occurs). Monitoring humidity levels according to the opening and closing of plant stomata can also help monitor the dehydration state of plants.

**FIGURE 7:**
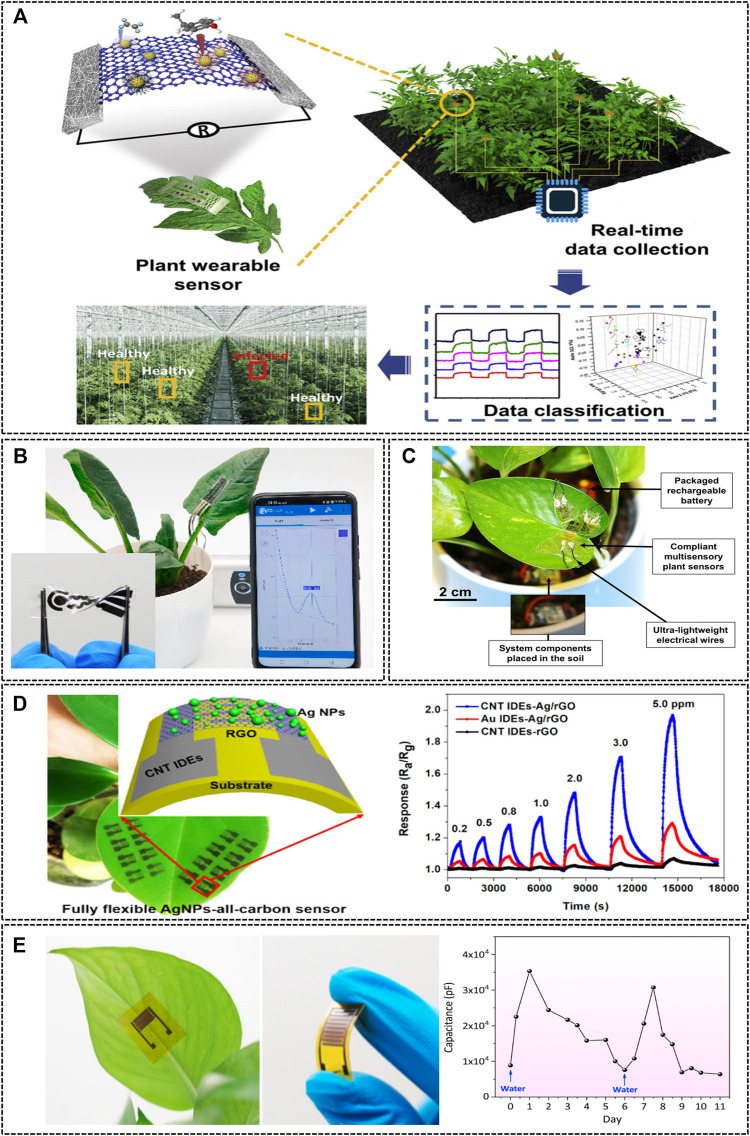
Plant-wearable sensors for determining plant physiological signals. **(A)** Late blight diagnosis in tomato through an abiotic stress monitoring system for sensing volatile organic compounds using a leaf-attached chemical sensor (Z. Li et al., 2021). Copyright ^©^ 2021 (Elsevier). **(B)** *In-situ* organophosphorus pesticide analysis on the surfaces of agricultural products (inset: the fabricated PDMS/LIG electrode) (F. Zhao et al., 2020). Copyright ^©^ 2020 (Elsevier). **(C)** An environmental plant sensor platform on the surface of a plant leaf (Nassar et al., 2018). Copyright ^©^ 2018 (Nature). **(D)** A gas sensor array attached to a real plant leaf, and schematic illustration of the flexible sensing device (left) and real-time response curves (right) (W. Li et al., 2018). Copyright ^©^ 2018 (American Chemical Society). **(E)** Plant humidity sensor for real-time monitoring; a humidity sensor attached to the back surface of a leaf (left) and the graphene oxide-based sensor (middle), and the capacitance response to drought stress (right) (Lan et al., 2020). Copyright ^©^ 2020 (Elsevier).

Continuously monitoring the growth requirements of crop plants is essential for increasing yields, especially in harsh environments. Recently, plant-wearable sensors have emerged in the field of plant health monitoring. These devices can monitor plant health conditions in real time, and can help detect diseases or abnormalities in plants at an early stage (Qu et al., 2021). For instance, plant leaves release volatile organic compounds (VOCs) when infected with *Phytophthora infestans*, and the presence of this disease can be monitored by detecting the released VOCs (Z. Li et al., 2021). A sensor was designed by integrating a graphene-based sensing material and a silver nanowire electrode on a stretchable substrate, and could detect diseases in tomato plants with >97% accuracy (Figure 7). Zhao et al. used LIG technology to developed a field analysis device for organophosphorus pesticides based on a 3-electrode system (F. Zhao et al., 2020). A stretchable and flexible LIG-based electrode was prepared with polydimethylsiloxane (PDMS). After being modified with an organophosphorus hydrolase (OPH), it could selectively detect the methyl parathione remaining on the surface of crops. OPHs hydrolyze methyl parathion to release p-nitrophenol, which electrochemically controls the catalytic reaction. In addition, the information was transmitted to a smartphone, which allowed real-time monitoring of the data. Nassar et al. developed an ultra-lightweight plant-wearable sensor with ultra-low power requirement (Nassar et al., 2018). The 3D-based stretchable plant-wearable sensor continuously monitors the local humidity and temperature on the leaf surface. Moreover, an innovative plant-wearable device with a 3D-printed origami-based plant copter design was used to improve plant-wearable sensors. Li et al. developed a device that directly prepared a target object by introducing an atomized gas sensor array using a silver nanoparticle (AgNP)–all-carbon hybrid nanostructure (W. Li et al., 2018). Reduced graphene oxide (rGO) decorated with AgNPs was used as a sensing layer on a metal single-star carbon nanotube as a conductive electrode base, and this sensor detected a low NO_2_ concentration (0.2 ppm) at room temperature. Moreover, Lan et al. designed a flexible and wearable humidity sensor that uses laser-induced graphene interdigital electrodes (Lan et al., 2020). The highly flexible graphene oxide (GO)-based humidity sensor could be attached to the surface of a plant leaf without physical damage, and using GO to detect humidity ensured high sensitivity (3,215.25 pF/% RH) and long-term stability (variation less than ±1%). As mentioned above, plant-wearable sensors with good flexibility can be attached to leaves or stems without causing physical damage, thus enabling real-time and long-term monitoring. Therefore, these plant-wearable sensors are expected to show excellent performance as next-generation electronic devices for PA.

Multi-measurement sensors can simultaneously monitor multiple external signals and indicate the health conditions of plants. However, the existing multi-measurement sensors only detect microclimatic factors such as temperature and humidity in the vicinity of plants, and systematic monitoring using multi-measurement sensors has limitations (Table 4). Therefore, in the future, it is essential to focus on the development of multi-measurement sensors that monitor plant biological signals and correlate them with environmental signals to elucidate plant physiological conditions. As discussed above, various parameters can be used for monitoring the overall health of plants. However, these should be integrated with each other, and future plant sensors should include sensor arrays capable of multiple measurements. Therefore, it is essential to improve the selectivity of biomarkers or catalysts that selectively react with active target molecules (Segev-Bar et al., 2017). In addition, it should be possible to simultaneously monitor plant responses to abiotic and biological stresses by combining existing sensors—such as temperature, humidity, and pH sensors—with an electrochemical measurement system. This would make it possible to predict and prevent a decrease in crop yield due to plant diseases and stress. Therefore, it is important to develop practical applications for multi-array and multi-measurement plant sensor systems with high sensitivity and selectivity.

**TABLE 4:** Multi-detection plant sensors.

System components	Sensing materials	Detection signals	Applications	References
Humidity/temperature/light sensor	ZIS, CNT	Resistance, Photoresistor, Thermistor	Plant health monitoring	Lu et al. (2020)
Hydration/strain/light/temperature sensor	PI, Cu, silicon-based phototransistor	Impedance, thermistor, piezoresistance, photoresistor	Plant physiology and microclimate	Zhao et al. (2019)
Humidity/UV sensor	p-doped pproDOT	Capacitance, impedance, phase	Tissue damage monitoring	Kim et al. (2019)
Humidity/temperature sensor	Titanium/gold	Capacitance, thermistor	Microclimate monitoring around plant	Nassar et al. (2018)

ZIS: ZnLn_2_S_4_; PI: polyimide; p-doped pproDOT: p-doped poly (3,4-propylenedioxythiophene).

## 3 Critical Issues

Over the past few decades, PA has been used to reduce costs, improve crop yields, and protect the environment. However, the adoption of these novel technologies by farmers is still limited due to the reasons discussed henceforth.

### 3.1 Robustness of Sensors

In the agricultural field, it is important for sensors to exhibit long-term stability under harsh environmental conditions (such as large fluctuations in temperature and humidity) and resist mechanical aging due to direct sunlight, rain, wind, and exposure to chemicals (such as pesticides). One, to improve the durability of sensors, it is necessary to incorporate a variety of materials. Research on the durability of plant sensors is still in its infancy, and a shift in the technologies used in human medicine may provide insights into improving the durability of plant sensors. The other, we need to develop a next-generation electrochemical sensor that must include a compact and ultra-small structure with greater rigidity, corrosion resistance, and an encapsulation layer with excellent waterproof performance.

### 3.2 Data Accumulation and Management

At present, the most important factor in agriculture is the insight into growing crops that farmers gain from decades of experience. Big data—a key element of the cultivation data management system—is a technology that seeks to replace farmers’ insights. Big data provides precise recommendations to growers, so that even beginners in agriculture can easily harvest crops according to the information provided, regardless of the maturity of the technology. However, there is no specific method for obtaining such crucial big data as soon as possible. Generally, big data in agriculture are obtained through interviews with numerous farmers. However, these methods are considerably labor- and time-consuming. Therefore, there is an urgent need to develop the technology to collect agronomic data in a prompt and accurate manner using sensors and information communication technologies.

Typically, big data for PA have been obtained using physical sensors to measure characteristics such as meteorological phenomena and soil moisture, pH, temperature, and electrical conductivity. Recently, data from image sensing by drones or satellites have been used to judge whether crop growth or yield can be predicted, and to decide whether a crop is infected with pests or diseases (Mogili and Deepak, 2018). High-resolution images of crops are obtained from the drone and then further processed to extract information, which is then used to make decisions and predictions. However, image sensing is insufficient for precise prescriptions, as it relies on a consequential point of view and judges the degree of growth of crops as a result of observing the outward appearance of crops.

PA involves data collection and processing for several parameters related to crop health, such as moisture, temperature, and nutrients. Thus, PA allows farmers to know exactly what parameters are needed to grow healthy crops, what the requirements are at any given point in time, and which parameters require attention. This requires gathering vast amounts of information from a variety of sources, such as nutrients in the soil and sap, the presence of pests and diseases, the chlorophyll content of plant leaves, and surrounding weather conditions. This collected information needs to be analyzed to generate agronomic recommendations by specialists. For instance, when considering the developmental stages of a plant, the size and color of a plant’s leaves can reveal deficiencies or excesses of nutrients. However, the phenotype of a plant cannot accurately determine which element is deficient, and visual examination only provides a rough estimate. Therefore, we need a technique that can use the plant phenotype to identify the degree of deficiency or excess of a specific nutrient. This technology includes an ion sensor that detects ions (nutrients) in a crop sap or hydroponic culture medium, and these nutrient data are combined with the weather conditions and the characteristics and nutrient distribution of the soil in which the plants are located. All this information is then used to determine exactly when and how much of a specific fertilizer should be applied to that plant. Consequently incorporating the ion sensor and an electrochemical technique as a data collection method for PA will provide be a breakthrough method for accurate prescriptions for future crop cultivation. Moreover, a multi-measurement system equipped with electrochemical sensors (to measure chemical and physical parameters) would form an integrative approach to providing comprehensive prescriptions.

### 3.3 Hardware Costs

PAs rely primarily on hardware such as sensing devices, wireless nodes, and automated systems, which are used to monitor the overall plant growth status in real time. In particular, these agricultural sensors have multiple limitations, including a high level of development, maintenance, and deployment cost. Agricultural management is mostly performed on small-scale arable land; therefore, it is necessary to supply inexpensive hardware components and sensors. To solve these problems, it is necessary to develop a low-cost multi-measurement sensing device that can measure several parameters simultaneously.

### 3.4 Environmental Effects

Agricultural activities account for 70% of water intake worldwide, and are considered the main cause of water pollution. (Moss, 2008). The main causes of pollution due to agricultural activities include nutrients, pesticides/herbicides, and salts. High rates of fertilizer use in crop cultivation cause not only soil pollution, but also water pollution (F. Zhao et al., 2020), (Wu et al., 2013). In addition, the abuse of these toxic substances can have a significant impact on biodiversity by destroying the ecosystem food chain. Therefore, the development of materials and systems that are less harmful to people and the environment—and that can obtain effective results when used in small amounts—has recently attracted attention.

A representative eco-friendly agricultural method is the closed hydroponic system (Hosseinzadeh et al., 2017). Hydroponics is a method of growing crops with a nutrient solution without using soil (Son et al., 2020), and presents the advantages of increasing production and producing high-quality crops. However, the waste nutrient solution causes environmental problems, as it causes soil pollution and the eutrophication of water systems (as described above). Therefore, closed hydroponic cultivation systems that reuse waste nutrient solutions are attracting attention as a new method for reducing agriculture-related environmental pollution. However, to reuse waste nutrient solution, several factors still toned to be considered, such as a sterilization facility for the spent nutrient solution, optimization technology for maintaining nutrients in the nutrient solution, and the establishment of an automatic circulation system. Therefore, the development of a closed hydroponic system—an eco-friendly agricultural cultivation method—should continue in the future.

### 3.5 Self-Powered Sustainable Agriculture System

Advances in various sensing techniques have enabled automated plant growth monitoring. However, developing sustainable power supplies for the electronic devices for sensing remains a challenge (Lan et al., 2021). To date, various types of sensing devices have been introduced that are fixed to the soil or directly attached to plant leaves or stems when monitoring environmental conditions or plant health. Additionally, the convergence of the IoT and sensing systems has made improved the automation of systems for monitoring and sensing information. On average, vegetables are harvested every 3–4 months, and the sensing device (including the battery) can operate for at least one cycle. However, a sustainable power supply for these electronic devices remains a fundamental problem. Currently, traditional batteries are used as temporary power sources, and must be replaced or recharged periodically, which complicates their maintenance and causes environmental problems. To overcome these issues, researchers have proposed the idea of a smart farm that uses renewable energy and utilizes surplus renewable energy for the agricultural field (Lin et al., 2021). An efficient energy consumption system (reactive energy utilization technology) was used to predict the optimal scenario that minimizes the total power loss with the lowest voltage in each scenario. Thus, a system that introduces renewable energy into the agricultural sector will provide an opportunity to expand the possibilities of sustainable precision agriculture.

## 4 Discussion and Conclusion

We systematically reviewed the major developments and applications of electrochemical plant sensors, and discussed the future prospects of PA. We emphasize the importance of sensors to monitor *in vivo* parameters such as nutrient concentration, hormone levels, and plant stress factors. Moreover, we focus on the need for plant-wearable sensors and multi-sensing systems in PA. In particular, the monitoring of phytochemicals has been emphasized more than the physical growth results of crops. The operating mechanism and performance of these electrochemical sensors are presented, and the applicability of each sensor in PA is discussed. Finally, we review some critical issues that obstruct the stable expansion of PA and the potential prospects for their resolution. Based on our comprehensive review, we believe that the crucial focus for the future of PA should be on the application of electrochemical multi-sensors to monitor plant health. The continuous collection of plant growth and environmental data in real time requires the development of a multi-measurement sensing platform that can be deployed on a large scale. The excellent performance, miniaturization, low cost, and environment-friendly characteristics of electrochemical plant sensors are expected to help upgrade the detection systems for PA. This review shed light on data acquisition strategies and novel plant sensors that can provide appropriate prescriptions in PA, and presents the future prospects for research on data agriculture systems. We believe that multi-measurement plant sensor systems will move from the laboratory to practical use in agriculture in the future, and we hope that this review will enable more researchers to conduct research on plant sensor systems for PA and contribute to the new field of smart farms.
